# NIL10: A New IL10-Receptor Binding Nanoparticle That Induces Cardiac Protection in Mice and Pigs Subjected to Acute Myocardial Infarction through STAT3/NF-κB Activation

**DOI:** 10.3390/pharmaceutics14102044

**Published:** 2022-09-26

**Authors:** Laura Tesoro, Ignacio Hernández, Rafael Ramírez-Carracedo, Javier Díez-Mata, Nunzio Alcharani, Beatriz Jiménez-Guirado, Karina Ovejero-Paredes, Marco Filice, Jose Luis Zamorano, Marta Saura, Carlos Zaragoza, Laura Botana

**Affiliations:** 1 Unidad de Investigación Cardiovascular, Departamento de Cardiología, Hospital Ramón y Cajal (IRYCIS), Universidad Francisco de Vitoria, 28034 Madrid, Spain; 2 Centro de Investigación Biomédica en Red de Enfermedades Cardiovasculares (CIBERCV), Instituto de Salud Carlos III (ISCIII), 28029 Madrid, Spain; 3 Departamento de Química, Facultad de Farmacia, Universidad Complutense (UCM), 28040 Madrid, Spain; 4 Centro de Investigación Biomédica en Red de Enfermedades Respiratorias (CIBERRES), Instituto de Salud Carlos III (ISCIII), 28029 Madrid, Spain; 5 Centro Nacional de Investigaciones Cardiovasculares (CNIC), 28029 Madrid, Spain; 6 Departamento de Cardiología, Hospital Universitario Ramón y Cajal (IRYCIS), 28034 Madrid, Spain; 7 Unidad de Fisiología, Departamento de Biología de Sistemas, Facultad de Medicina, Universidad de Alcalá, 28871 Madrid, Spain

**Keywords:** acute myocardial infarction, interleukin-10, nanoparticles, STAT-3, NF-κB

## Abstract

(1) Background: Early response after acute myocardial infarction (AMI) prevents extensive cardiac necrosis, in which inflammation resolution, including expression of anti-inflammatory interleukin-10 (IL-10), may play a key role. (2) Methods: We synthesized NIL10, a micelle-based nanoparticle, to target IL-10 receptor in mice and pigs subjected to AMI. (3) Results: Administration of NIL10 induced cardiac protection of wild-type and IL-10 knockout mice and pigs subjected to AMI. Cardiac protection was not induced in IL-10-receptor null mice, as shown by a significant recovery of cardiac function, in which inflammatory foci and fibrosis were strongly reduced, together with the finding that resolving M2-like macrophage populations were increased after day 3 of reperfusion. In addition, anti-inflammatory cytokines, including IL-4, IL-7, IL-10, IL-13, IL-16, and IL-27 were also elevated. Mechanistically, NIL10 induced activation of the IL-10 receptor/STAT-3 signaling pathway, and STAT3-dependent inhibition of nuclear translocation of pro-inflammatory NF-ĸB transcription factor. (4) Conclusions: Taken together, we propose using NIL10 as a novel therapeutic tool against AMI-induced cardiac damage.

## 1. Introduction

Cardiovascular diseases, including acute myocardial infarction (AMI), are the most frequent cause of death in the world [1]. AMI remains the commonest origin of heart failure (HF), which conditions long-term life quality and may result in the death of the patient [2,3,4].

Following an ischemic event, the lack of oxygen and nutrients in the heart triggers an inflammatory response. Necrotic cardiomyocytes release signals that stimulate innate immune signaling, then cell mobilization to the damaged area begins, and, finally, anti-inflammatory signaling leads to the replacement of necrotic myocardial tissue by the formation of a fibrotic scar [5]. Even though inflammation is a critical component of tissue healing, currently, more contributions point towards a prolonged inflammatory response compromising myocardial structure and function due to an adverse left ventricle remodeling, causing HF [6,7,8]. Therefore, targeting the inflammatory response may represent a promising strategy to prevent adverse outcomes after AMI [9].

Macrophages play homeostatic and immune control roles in myocardial remodeling throughout the process of inflammation [10] and polarize according to signals from their environment [11]. Altogether, in response to stimuli such as bacterial lipopolysaccharide (LPS), interferon gamma (IFN-γ), or even hypoxic stimuli, M1-like activated macrophages secrete pro-inflammatory cytokines, tumor necrosis factor (TNF-α), granulocyte colony-stimulating factor (G-CSF), and granulocyte-macrophage colony-stimulating factor (GM-CSF) motif chemokine ligand (CXCL) as CXCL10, and matrix metalloproteinases (MMPs) as MMP-2, MMP-9, and MMP-13. This population expands at the site of inflammation and change their phenotype, serving as the first line of defense within hours–days. M2-like-resolving macrophages induced by anti-inflammatory cytokines, such as IL-4, IL-13, and IL-10, are capable of inducing fibroblast-mediated extracellular matrix production, cell proliferation, and angiogenesis, leading to the repair of damaged tissues [12,13]. Mechanisms to promote polarization towards M2-like state, including backward prevention to M1-like repolarization, are essential to avoid adverse cardiac remodeling, a process in which the anti-inflammatory IL-10 is mainly involved.

IL-10 binding to its receptor (IL-10R) [14] results in activation of receptor-associated tyrosine kinases of the Janus kinase family, JAK1 and TYK2, which in turn lead to phosphorylation of two tyrosine residues on the receptor. IL-10R is composed of two subunits; both have roles in signal transduction processes [15,16,17], which mainly induce anti-inflammatory responses, playing a key role in macrophage-mediated inflammation resolution. Phosphorylated IL-10R1 residues recruit STAT3 to the activated receptor, which causes the subsequent phosphorylation and activation of the latent transcription factor, phosphorylated-STAT3 [18,19].

The use of specific anti-IL-10 antibodies is a successful approach to show the relevance of IL-10 during hindlimb reperfusion after femoral artery ligation [20], chronic inflammation [21], certain types of cancer [22,23], and neuropathic pain [24]. Nevertheless, and to avoid possible antibody-induced immunogenicity responses, we designed lipid nanoparticles (NIL10) conjugated with IT9302 [25], a nanomeric peptide, homologous to the C terminus of IL-10, or a scramble peptide (NIL10SC) as a negative control, to evaluate its contribution to the resolution of inflammation following acute myocardial infarction in mice and pigs

## 2. Materials and Methods

### 2.1. Reagents and Equipment

Hematoxylin-eosin (HE), Masson trichrome, triphenyl tetrazolium chloride (TTC), Evans blue, and fetal bovine serum (FBS) were from Merck (St. Louis, MO, USA). Horseradish peroxidase (HRP)-conjugated antimouse secondary antibody and liquid 3,3′-diaminobenzidine (DAB) substrate were from Dako (Santa Clara, CA, USA). Anti-IL-10RA (ab225820) anti-MMP-9 (ab38898), anti-GAPDH (ab22555), and anti-β-Tubulin (ab6046) antibodies were from Abcam (Cambridge, UK). Anti-NF-κB (sc-372) and anti-NOS2 (sc-651) antibodies were from Santa Cruz (Santa Cruz, CA, USA). Goat antirabbit IgG Alexa Fluor 488 secondary antibody (A11008), anti-P-IL10-RA (PA5-104991), 100 μm cell strainer (08-771-19), ACK lysing Buffer (A1049201), anti-CD68 (11-0689-42), and anti-CD206 (12-2069-42) were from Thermo Fisher Scientific (Waltham, MA, USA). Anti-STAT3 (79D7), anti-P-STAT3 (Y705, D3A7), and anti-P-IκB-α (S32, 14D4) antibodies were from Cell Signaling (Danvers, MA, USA). Proteome Profiler Mouse Cytokine Array Kit (ARY006) and Proteome Profiler Human Cytokine Array Kit (ARY005B) were from R&D Systems (Minneapolis, MN, USA). Ketamine was from Pfizer (New York, NY, USA), isoflurane was from Abbvie (North Chicago, IL, USA), propofol was from Fresenius (Bad Homburg, Germany), fentanyl was from Kern Pharma (Madrid, Spain), diazepam was from Roche (Basel, Switzerland), and the amiodarone was from Sanofi Aventis (Gentilly, France).

### 2.2. Peptide and Nanoprobe Composition

Peptide composition of IT9302 and scrambled: IT9302: CAYMTMKIRN. IT9302-scrambled: CANYRMITKM.

**Figure 1:**
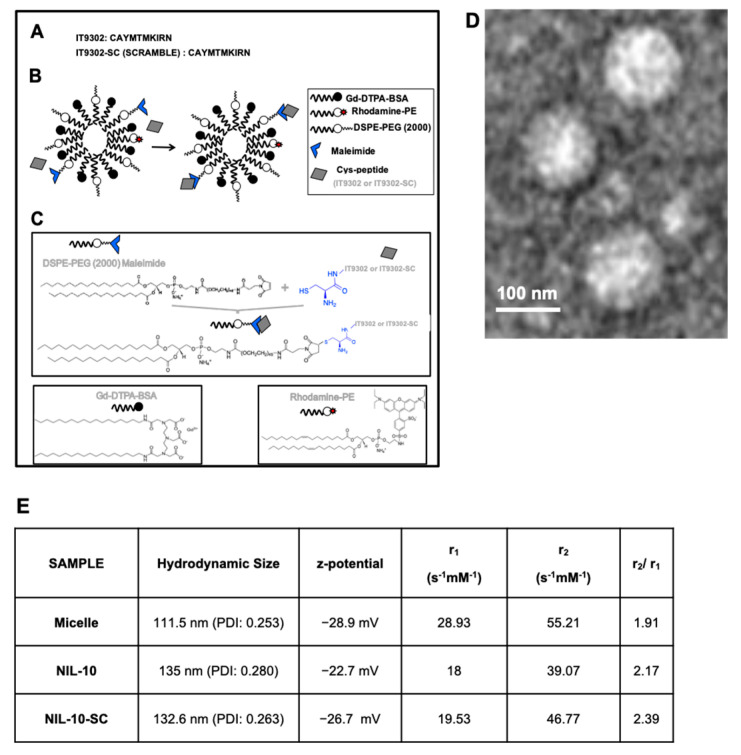
Nanoparticle composition and properties. (**A**–**C**) Nanoparticle structure. (**D**) Transmission electron microscopy visualization of NIL10. (**E**) Physicochemical properties: Z-potential, longitudinal and transversal relaxivities of NIL10 and NIL10SC nanoparticles.

The paramagnetic nanoparticles were prepared by the lipid film hydration method. In brief, a film was prepared by rotary evaporation of Gd-DTPA-bis (GdDTPA-BSA), 1,2-distearoyl-sn-glycero-3-phosphoethanolamine-N-[methoxy (polyethylene glycol)-2000] (DSPE-PEG2000), DSPE-PEG2000-maleimide, and Rhodamine-PE, in a molar ratio 50:39:1:10, dissolved in a mixture of chloroform/methanol (4:1 *v*/*v*). The lipid film was hydrated with HEPES buffer, pH 6.7, and 150 mM NaCl, and the solution was rotated at 65 °C for 1 h. IT9302 and the corresponding scrambled peptides were modified by adding a terminal cysteine residue (Figure 1) to bind to the maleimide moiety at a molar ratio of 1:40 (micelle: peptide). Uncoupled peptides were separated with centrifuge concentrators of 100 kDa. The number of peptide molecules bound per micelle was calculated by HPLC as described [26], estimating 4 ± 2 and 5 ± 1 as the number of peptides present in NIL10 and NIL10SC, respectively. Physical and chemical properties, including Zeta-potential, nanoparticle size (calculated as hydrodynamic diameter with dynamic laser light scattering (DSL, Malvern Zetasizer)), nanoparticle morphology (visualized with transmission microscopy (TEM)), and longitudinal and transverse relaxivities, were calculated. Longitudinal relaxivities were evaluated by using inversion recovery sequences with 15 inversion times. Transverse relaxivities were calculated from spin-echo images and different echo times. In both cases, a series of images with different T1 and T2 weighting were generated. The r1 and r2 values were estimated from the slope of longitudinal and transverse relaxation rates vs. Gd-DTPA or nanoparticle.

### 2.3. Animal Studies

All the surgical procedures were performed in the Experimental Surgery Department of the Hospital Universitario La Paz in Madrid (Spain). The procedures conformed to the Guide for the Care and Use of Laboratory Animals published by the US National Institutes of Health (NIH Publication No. 85-23, revised 1985), the Animal Welfare Ethics Committee, the EU Directive on experimental animals (63/2010 EU), and the related Spanish legislation (RD 53/2013), PROEX 368-15 (mouse studies) and PROEX 052.4-22 (porcine studies).

#### 2.3.1. Porcine Model of Coronary Ischemia/Reperfusion

Twenty male Yorkshire pigs (30 ± 4 kg) were housed 1 week preceding the surgery to avoid unease or stress associated with the new environment. Prior to the surgical intervention, animals were anesthetized with 10 mg/kg intramuscular ketamine and 0.5 mg/kg midazolam. Anesthesia was induced by inhaled sevoflurane and maintained with continuous infusion of 2 mL/kg/h propofol, 50 µg/kg/h fentanyl, and 10 µg/kg/h diazepam. After intubation and mechanical ventilation with 100% oxygen saturation, 5000 IU of heparin and 2 mg/kg/h amiodarone was administered to avoid blood clotting of catheters and malignant cardiac arrhythmias, respectively. Preceding the complete occlusion, hearts were submitted to ischemic preconditioning by blocking the LAD for short periods (1, 3, and 5 min each). Ischemia/reperfusion was produced by occluding LAD for 45 min using a JL3 6F catheter and an angioplasty balloon. The complete myocardial ischemia was confirmed by ST-segment elevation. Then, the balloon was removed to reopen the artery. Animals received NIL10 or NIL10SC (0.1 mg/kg) 24 h postischemia. Blood was extracted before and after the procedure, 3 days postischemia, and at the final point. After 7 days of reperfusion, animals were sacrificed to extract the heart, spleen, kidney, lung, liver, and pancreas. A portion was included in 4% formalin and the rest was immediately frozen at −80 °C for protein analyses. 

#### 2.3.2. Murine Model of Coronary Ischemia/Reperfusion

Wild-type, IL-10 knock-out, and IL-10RA knock-out C57/BL6 male mice were anesthetized with 3% sevoflurane and oxygen inhalation with a flow rate of 0.4 L/min until loss of righting reflex. Endotracheal intubation was performed using an intubation cannula for artificial ventilation (tidal volume: 260 μL/stroke, ventilation rate: 130 strokes per minute). The fourth left intercostal space was opened and widened using mouse chest retractors. The left ventricle was then exposed, and the LAD was occluded for 30 min by using a 6–0 silk suture and 1 mm tube. Reperfusion was performed by ligation release, and after the procedure, the chest was closed, negative pressure restored, and the skin sutured. At the reperfusion time of 24 h, NIL10 was intravenously administrated. Animals were sacrificed on days 3 and 7 postprocedure to extract the organs and process the samples. NIL10SC-treated animals were included in the assays as control, in which the same procedure was performed.

### 2.4. Echocardiography

Vivid Q ultrasound system from GE Healthcare (General Electric, Chicago, IL, USA), equipped with a 1.9–4 MHz scan head was used to determine LV function. Parasternal long- and short-axis-view images of the heart were taken prior to the surgery, at the end of ischemia, and at the endpoint to determine LV function worsening and recovery. The parameters studied using the onsite software cardiac package were: systolic and diastolic interventricular septum thickness (IVS), systolic and diastolic left-ventricle internal diameter (LVID), systolic and diastolic left-ventricle posterior wall thickness (LVPW), left-ventricle ejection fraction (LVEF), left ventricle shortening fraction (LVFS), heart rate (HR), and cardiac output (CO). Data acquisition and analysis were performed by using the onsite cardiac software VividQ ultrasound equipment, and the same operator analyzed all samples to avoid interobserver biases.

### 2.5. Histology

Evans blue/TTC staining in pigs was performed as described [27]. In brief, 7 days after surgery, the LAD was re-occluded through balloon inflation at the same location as day 0. Then, a 5F fenestrated pigtail catheter was inserted through the second femoral artery and placed into the left ventricle to inject 200 mL of 5% Evans blue solution to distribute the compound across the entire cardiovascular system, excepting the blood-deprived area of the heart. After 2 min, the animal was sacrificed, and the heart was extracted and frozen at −20 °C for 24 h. Then, 0.8 cm heart slices were incubated in 1% TTC solution for 20 min at 37 °C and in 10% paraformaldehyde solution. The necrotic area was calculated in reference to the area at risk to avoid intervariability biasing.

Heart morphology was visualized by hematoxylin/eosin staining and collagen deposition was detected by Masson’s Trichrome staining both in mice and pigs.

### 2.6. Confocal Microscopy

Rhodamine containing NIL10 nanoparticles were visualized in 5 μm heart sections mounted in PBS buffer containing Hoechst for nuclei visualization by confocal microscopy. 

RAW 264.7 cell monolayers were incubated with anti-NFκB primary antibody diluted 1:500 in PBS 1.5% BSA for 1 h. They were then washed and incubated with the corresponding goat anti-rabbit IgG Alexa Fluor 488 secondary antibody diluted 1:1000 in PBS 1.5% BSA for 1 h. Cells were washed three times with PBS and mounted in PBS media containing Hoechst for nuclei visualization. Images were taken using a Leica TCS SP5 confocal microscope. At least three different fields per condition were obtained.

### 2.7. Immunoblotting

Protein lysates from healthy and necrotic areas of mouse and pig hearts were extracted with RIPA buffer to measure the levels of MMP-9, IL-10R, P-IL-10R, STAT3, and P-STAT3, while and P-IκB-α, and NOS2 were assayed in RAW247 cells. Proteins were separated by SDS-PAGE and transferred to PVDF membranes. Membranes were blocked with 3% BSA in 25 mM Tris, 150 mM NaCl, 0.05% Tween-20, pH 7.4 (T-TBS), washed and incubated with the corresponding primary antibodies diluted 1:1000 for one hour at room temperature. Membranes were subsequently washed 3 times with T-TBS and then incubated with horseradish peroxidase-conjugated secondary antibodies 1:3000 for an hour for detection of proteins by chemiluminescence. The levels of GAPDH and β-Tubulin were used as loading controls.

### 2.8. Blood Collection and Plasma Isolation

Animal blood samples were collected in sodium citrate (363086) and EDTA tubes (367861) (BD Vacutainer, Franklin Lakes, NJ, USA) from a retro-orbital bleed in mice or from femoral venous in pigs. Blood samples were centrifuged at 400× *g* for 10 min, and the plasma was stored at −20 °C.

### 2.9. Cytokine and Chemokine Determinations

We detected a total of 40 human cytokines, chemokines, and acute phase proteins simultaneously from the serum of pigs and mice at the times indicated, by using the Proteome Profiler Cytokine Array Kit (RD Systems, Minneapolis, MN, USA), a membrane-based antibody array for the parallel determination of the relative levels of selected human cytokines and chemokines, as described [9].

### 2.10. Single-Cell Suspension for Flow Cytometry

Whole mouse hearts were mashed in complete DMEM (10% FBS, penicillin/streptomycin) through a 100 μm cell strainer. ACK Lysing Buffer was added to the single-cell suspension and spun down at 350 g for 5 min. The ACK was washed out with 50 mL of washing buffer (1% FBS), and cells were incubated with 1:100 anti-CD68 and anti-CD206 antibodies. All flow cytometry samples were assayed in a MACSQuant Analyzer Flow Cytometer, analyzed with the MACSQuantify Software and results were graphed with the Graphpad Prism software package.

### 2.11. Statistical Analysis

All values were given as mean ± SD. Significance is reported at the 5% level. Whenever comparisons were made with common control, the significance of differences was tested by Dunnett’s modification of the *t*-test.

## 3. Results

### 3.1. NIL10 Improves Cardiac Function in Mice Subjected to IR

**Figure 2:**
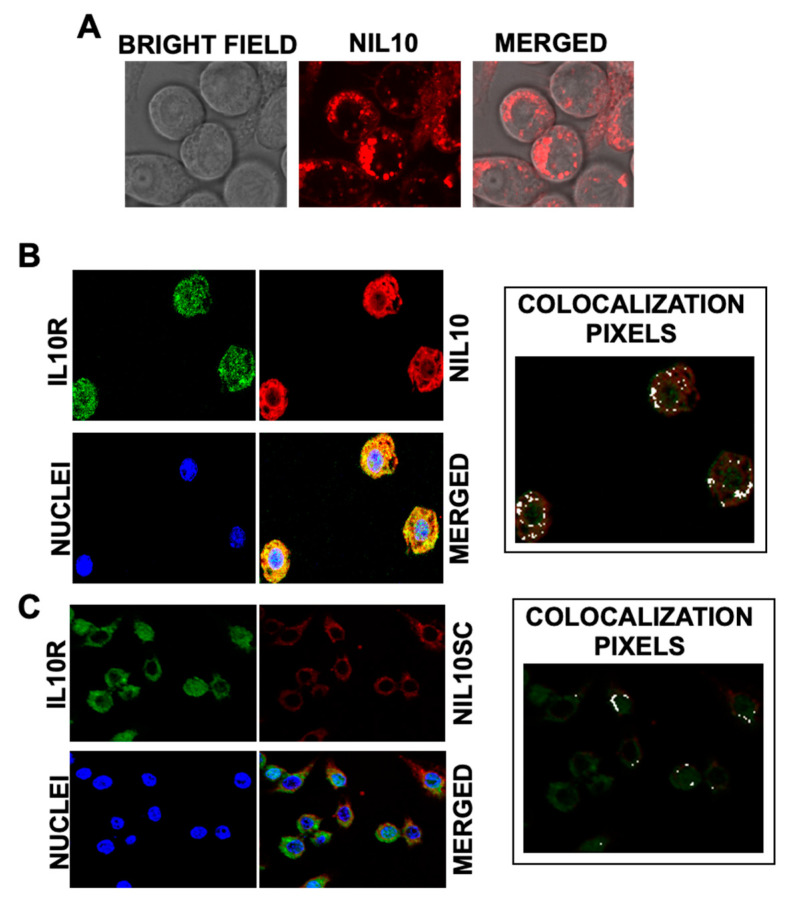
NIL10 binds to IL-10R in RAW 264.7 cells. (**A**) Bright-field and confocal microscopy sections of RAW 264.7 macrophages, detecting rhodamine (red) containing NIL10 nanoparticles. (**B**,**C**) Colocalization of NIL10 (**B**) (rhodamine, red) or NIL10SC (**C**) with IL-10R (FICT, green) in confocal microscopy sections of RAW 264.7 cells incubated with NIL10 and anti-IL-10R specific antibody. Merged panel show the co-localization of both signals. Nuclei were stained with Hoechst. Right panel: colocalization analysis with Image J co-localization plugin software, in which white dots correspond to co-localization (*n* = 3).

To test whether NIL10, a PEGylated nanoparticle conjugated with analog IL-10 peptide IT9302 (Figure 1A–C for physicochemical details), binds to the IL-10 receptor, we incubated RAW 264.7 macrophages with 1 μg/mL NIL10 (a nanoparticle conjugated with specific peptide IT9302) or NIL10SC (the same nanoparticle in which IT9302 was substituted by a scrambled peptide (see methods for details)), detecting rhodamine-containing nanoparticles by confocal microscopy (Figure 2A). Colocalization with the IL-10R (as detected by immunohistofluorescence (FITC, green)) was positive in cells incubated with NIL10 (rhodamine, red. Figure 2B), as detected by confocal microscopy (merged panel, yellow and colocalization pixels panel). No binding was found in cells incubated with NIL10SC, as NIL10SC is conjugated with a peptide that does not bind to IL-10R (Figure 2C). Cell viability was not affected at the dosage of NIL10 tested.

We intravenously administered NIL10 and NIL10SC at dosages of 0.1 mg/kg, 1 mg/kg, and 10 mg/kg, and analyzed the tissue distribution by confocal microscopy in the heart, liver, kidney, pancreas, and spleen of healthy animals at times 7, 14, 21, and 28 days after injection. We found a faint accumulation of NIL10 in the heart and kidney by day 7 after injection of 1 mg/kg and 10 mg/kg (Supplementary Figure S1). On the other hand, Kaplan–Meier curves of healthy mice injected with 1, 10, and 100 mg/kg indicated no mortality linked to the lowest dose, whereas the dosages of 10 and 100 mg/kg were lethal for 20% and 75% of animals, respectively, after 30 days of testing (Supplementary Figure S2).

**Figure 3:**
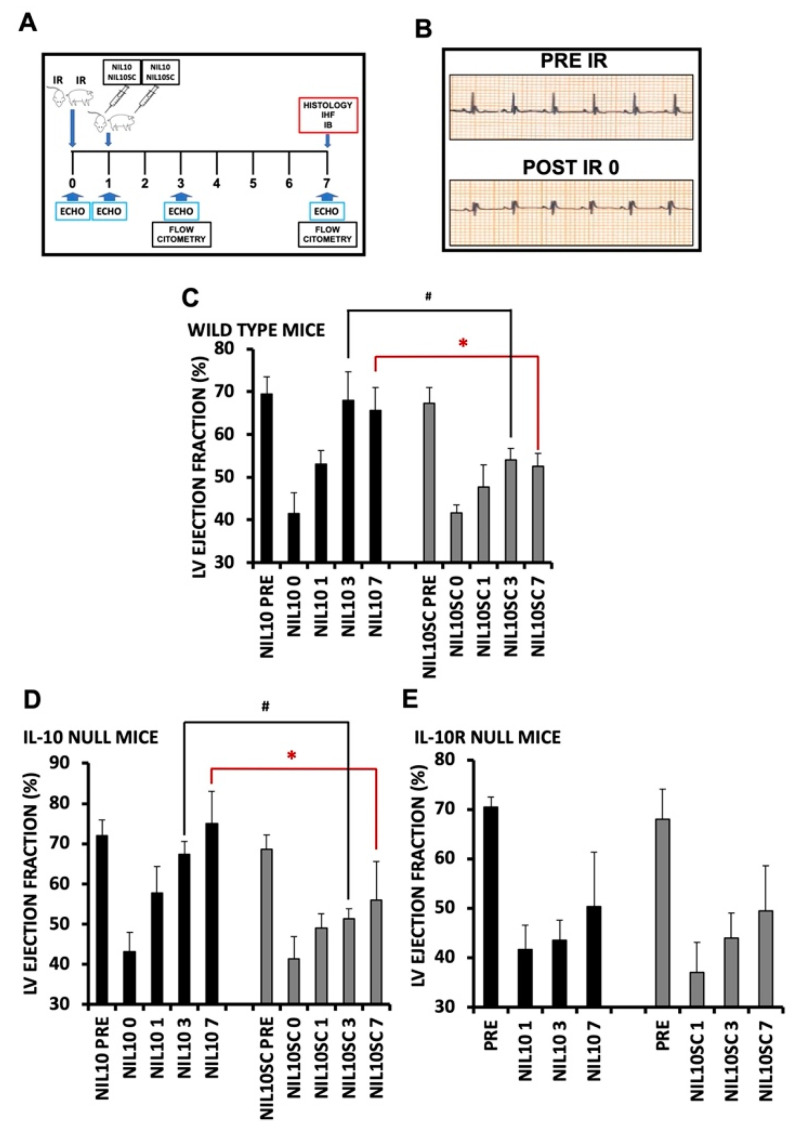
NIL10 induces cardiac protection in mice subjected to IR. (**A**) Graphical view of the procedures performed. (**B**) Representative electrocardiogram, showing ST-elevation after LAD occlusion. (**C**) Left ventricle ejection fraction (LVEF) of wild-type mouse hearts at the times indicated after IR and injected with NIL10 or NIL10SC as in A. *n* = 10 mice/group. Results are expressed as mean ± SD. * *p* < 0.03 NIL10 vs. NIL10SC day 7. # *p* < 0.05 NIL10 vs. NIL10SC day 3. (**D**) LVEF in IL-10 knockout mice. *n* = 10 mice/group. Results are expressed as mean ± SD. * *p* < 0.01 NIL10 vs. NIL10SC day 7. # *p* < 0.05 NIL10 vs. NIL10SC day 3. (**E**) LVEF in IL-10 receptor knockout mice. *n* = 10 mice/group. Results are expressed as mean ± SD.

After selecting a dosage of 1 mg/kg, we proceeded to inject 1 mg/kg of either NIL10 or NIL10SC into mice 24 h after IR (Figure 3A,B). The result was that NIL10 improved the LVEF by days 3 and 7 after IR when compared to NIL10SC (41.5% ± 4.33 vs. 68% ± 7.02 and 65.6% ± 5.11, respectively. Figure 3C). Proof of concept was obtained by performing the same assay in IL-10 null mice, in which NIL10 also exhibited a significant degree of cardioprotection; administration of NIL10SC did not show any improvement (Figure 3D). By contrast, in IL-10-receptor-deficient mice subjected to IR, NIL10 had no effect (Figure 3E), suggesting that NIL10 induces cardiac protection through activation of the IL-10 receptor signaling pathway.

### 3.2. Administration of NIL10 Reduces Necrosis and Fibrosis in Mouse Hearts Subjected to IR

**Figure 4:**
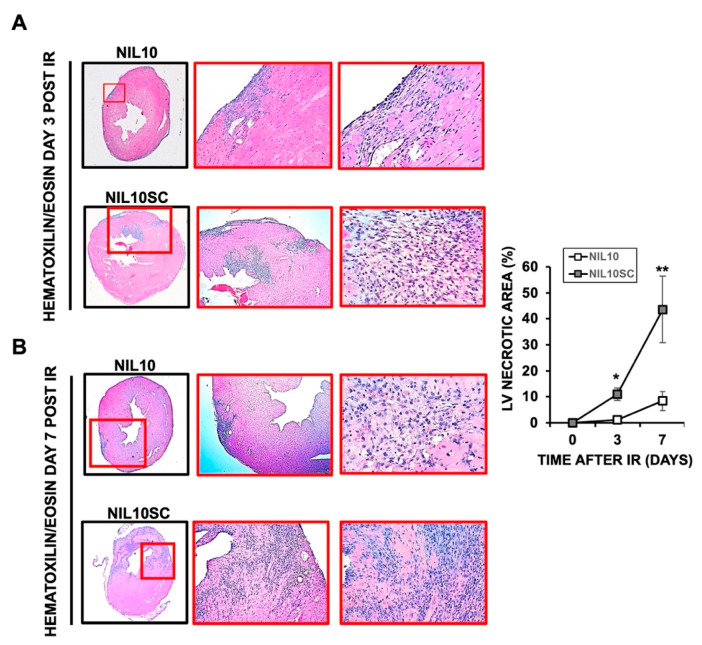
Hematoxylin and eosin staining of mouse hearts. Heart sections of mice subjected to IR and injected with NIL10 or NIL10SC, and collected by days 3 (**A**) or 7 (**B**) after IR. *n* = 10 mice/group. From left to right: 2×, 20×, 40×. * *p* < 0.05 D3; ** *p* < 0.01 D7.

**Figure 5:**
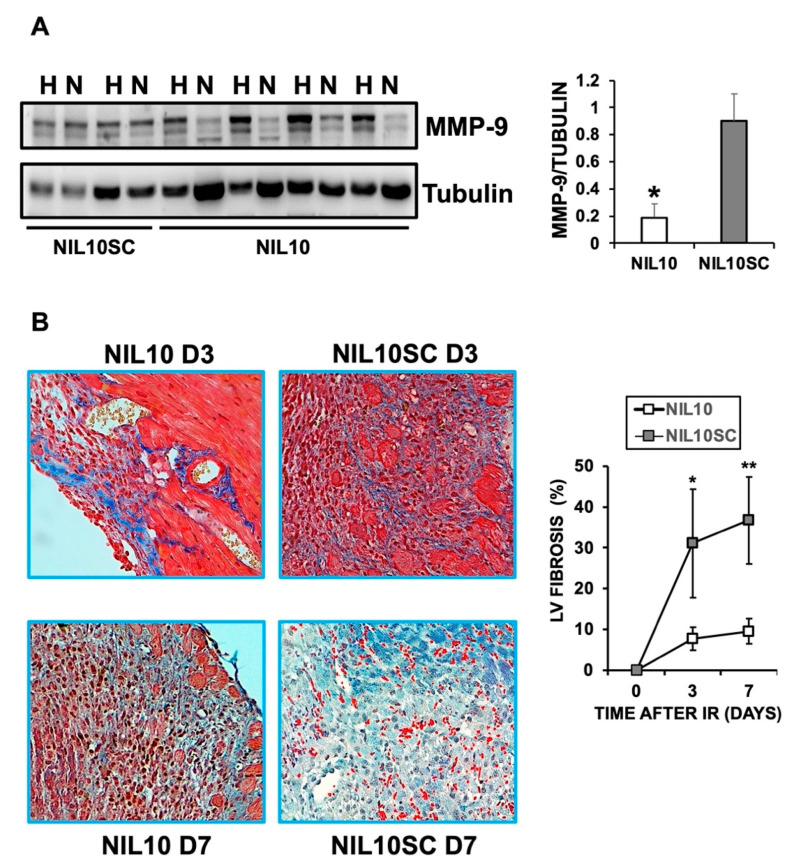
NIL10 reduce heart fibrosis in response to IR. (**A**) Representative immunoblot of MMP9 from healthy (H) or necrotic (N) areas of mouse hearts after 7 days of IR and injected with NIL10 or NIL10SC. *n* = 6 mice/group. Mean ± SD. * *p* < 0.001 NIL10 vs. NIL10SC necrotic areas. (**B**) Masson trichrome staining of heart sections from mice injected with NIL10 or NIL10SC, after 3 or 7 days post-IR (*n* = 6 mice/group). * *p* < 0.05 D3; ** *p* < 0.03 D7.

To shed light on the cardioprotective effect of NIL10, Hematoyilin/eosin (H/E) staining of heart sections, isolated on days 3 and 7 after IR, showed a significant reduction in the inflammatory foci and the extension of myocardial necrosis in response to NIL10 when compared to NIL10SC (Figure 4). Likewise, the levels of the necrosis marker, matrix metalloproteinase 9 (MMP9), were markedly reduced in the necrotic areas of NIL10 mice (Figure 5A), including the extension of fibrosis, whereas in the NIL10SC group, fibrotic lesions were widespread in the hearts by day 7 after IR (Figure 5B).

### 3.3. NIL10 Improves Cardiac Function in Pigs Subjected to IR

**Figure 6:**
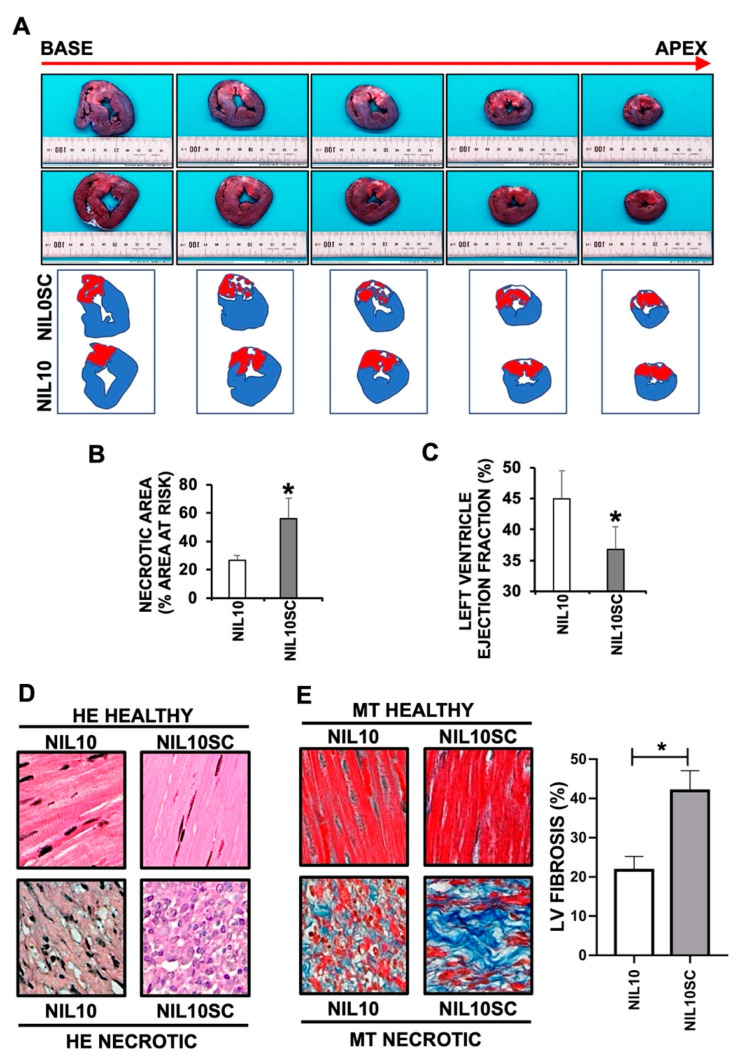
NIL10 induces cardiac protection in pigs subjected to IR. (**A**) Evans blue/TTC staining (see methods for details) of pig heart sections (from apex to base) after 7 days of IR, showing healthy tissue (blue), the area at risk (red) and the necrotic areas (white). (**B**) Measurement of necrotic areasof the hearts represented as a percentage with respect to the area at risk (Mean ± SD. * *p* < 0.001 NIL10 vs. NIL10SC, at day 7 post-IR). (**C**) Left ventricle ejection fraction of hearts from pigs injected with NIL10 or NIL10SC after 7 days of IR (Mean ± SD. * *p* < 0.001 NIL10 vs. NIL10SC). (**D**) Hematoxylin and eosin staining of healthy and necrotic heart sections from pigs injected with NIL10 or NIL10SC after 7 days of IR. (**E**) Left. Representative Masson trichrome staining of the same hearts as in D. Right. Graphical representation of fibrosis (Mean ± SD; * *p* < 0.03 NIL10 vs. NIL10SC; *n* = 6 pigs/group).

To test whether NIL10 may also induce cardiac protection in large animals, we injected 1 mg/kg of either NIL10 or NIL10SC into pigs subjected to IR by angioplasty balloon inflation of the left anterior descending coronary artery (Figure 3A). As shown by Evans blue/TTC staining of left ventricle sections (Figure 6A), we found a significant reduction in the necrotic area between the NIL10 and NIL10SC groups by 47% after 7 days of IR (NIL10SC 55 ± 7.34 vs. NIL10 26.48 ± 4.32) (Figure 6B), which contributes to explain the improvement in cardiac function as evidenced by the recovery of LVEF in animals injected with NIL10 (Figure 6C). As in mice, HE and Masson’s trichrome staining of heart sections from pigs injected with NIL10 indicated a marked reduction of myocardial necrosis (Figure 6D) and fibrosis (Figure 6E) when compared with the NIL10SC group.

### 3.4. NIL10 Has an Impact on Macrophage Polarization

**Figure 7:**
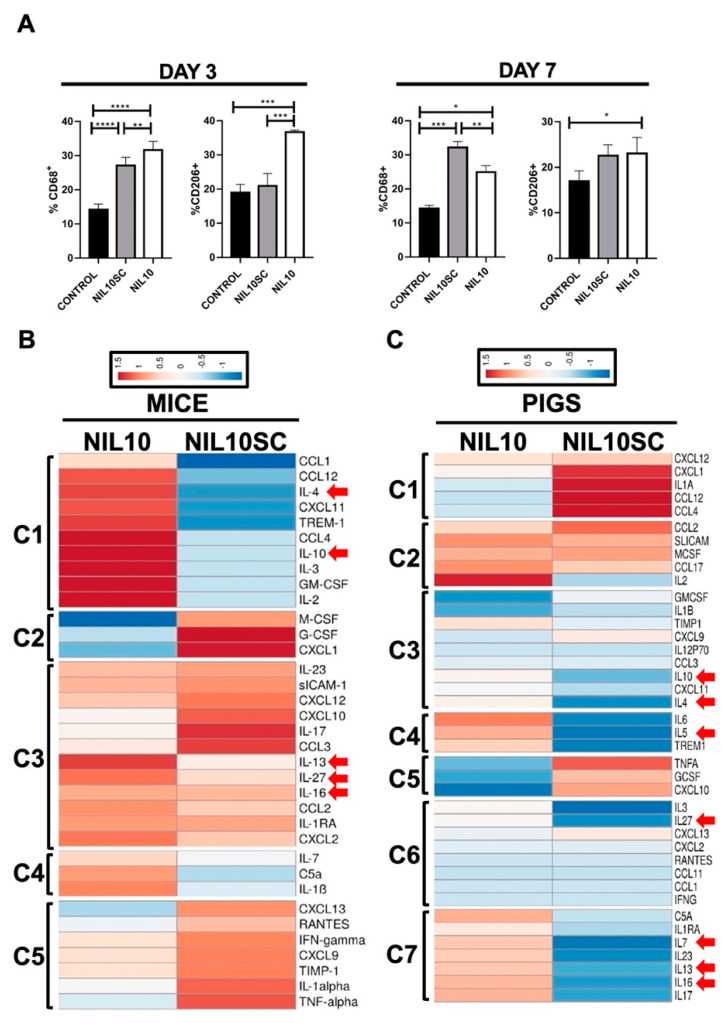
NIL10 induces M2 macrophage polarization in the hearts of mice subjected to IR and induces the expression of resolving cytokines in mice and pigs. (**A**) Flow cytometry analysis of macrophage populations in mouse hearts after 3 and 7 days of IR. The percentage of the M2 CD68+/CD206+ population was selected from the necrotic and risk areas of the hearts. (*n* = 6 mice/group). Differences between groups were compared using one-way ANOVA. * *p* < 0.05, ** *p* < 0.01, *** *p* < 0.001, **** *p* < 0.0001. (**B**) Clustered heat map of the differentially expressed cytokines in mice. (**C**) Clustered heat map of the differentially expressed cytokines in pigs. Red arrows indicated anti-inflammatory cytokines.

The difference in the number and intensity of inflammatory foci led us to study whether NIL10 could have an impact on macrophage polarization. M2-resolving macrophages increased in NIL10 and NIL10SC at days 3 and 7, respectively (Figure 7A), suggesting that NIL10 accelerate macrophage polarization, and further supports the severity of inflammation found in the group of NIL10SC.

We also assessed the level of 40 cytokines and chemokines in plasma collected from mice and pigs after 3 days of IR (Supplementary Figure S3). We identified differences in the expression of a significant number of cytokines, highlighting the presence of four mouse (5 pig) expression clusters [28], depending on whether the animals have been injected with NIL10 or NIL10SC. This highlighted that, in both species, we identified a marked increase in the expression of anti-inflammatory cytokines IL-4, -10, -13, -16, and -27, together with IL-5, and IL-7 in pigs, in response to NIL10 administration (Figure 7B,C). Taken together, these results suggest that NIL10 may induce cardiac protection by, at least, macrophage polarization towards inflammation resolution.

### 3.5. NIL10 Induces Phosphorylation of STAT3 in Pigs Subjected to IR

Cardiac exposure to ischemia triggers activation of specific pro-inflammatory transcription factors, of which NF-κB plays a major role [29]. Polarization of immune cells through an inflammatory resolving state implies the activation of specific signaling cascades, highlighting the contribution of IL-10-induced JNK signaling pathway that activates STAT3 transcriptional regulation of anti-inflammatory pathways, including prevention of NF-κB nuclear translocation [30]. 

**Figure 8:**
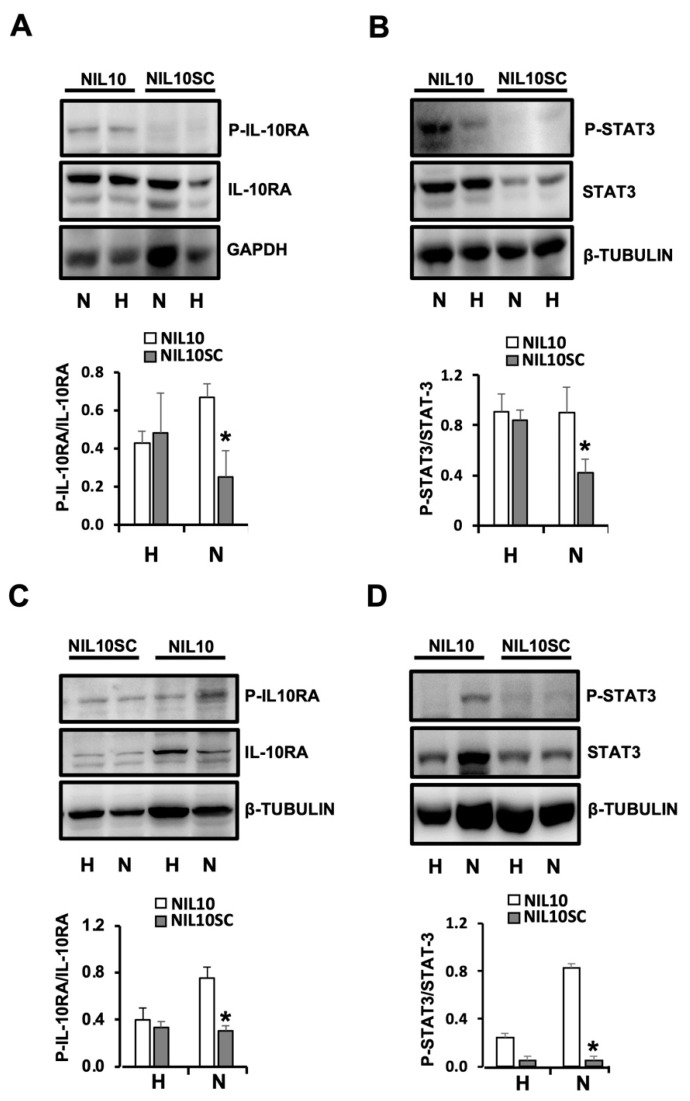
NIL10 induces phosphorylation of IL10RA and STAT3. (**A**) Immunoblot detection of P-IL10RA (**upper**), and total IL-10-RA in the healthy (H) and necrotic (N) areas of mouse hearts at day 7 after IR (*p* < 0.04 NIL10 vs. NIL10SC necrotic areas). (**B**) Immunoblot detection of P-STAT3, and total STAT3 in the same protein extracts. *n* = 6 mice/group. Results are expressed as Mean ± SD (*p* < 0.05 NIL10 vs. NIL10SC necrotic areas). (**C**) Immunoblot detection as described in A. of pig hearts at day 7 after IR (*p* < 0.05 NIL10 vs. NIL10SC necrotic areas). (**D**) Immunoblot detection of P-STAT3, and total STAT3 in the same protein extracts. *n* = 6 pigs/group. Results are expressed as Mean ± SD (* *p* < 0.005 NIL10 vs. NIL10SC necrotic areas).

NIL10 led to an accumulation of phosphorylated IL-10 receptor subunit IL-10RA and phospho-STAT3 in mice (Figure 8A,B) and in pigs subjected to IR (Figure 8C,D), indicative that NIL10, but not NIL10SC, induces receptor–agonist complex activation through the IL-10/JNK-STAT3 signaling pathway.

**Figure 9:**
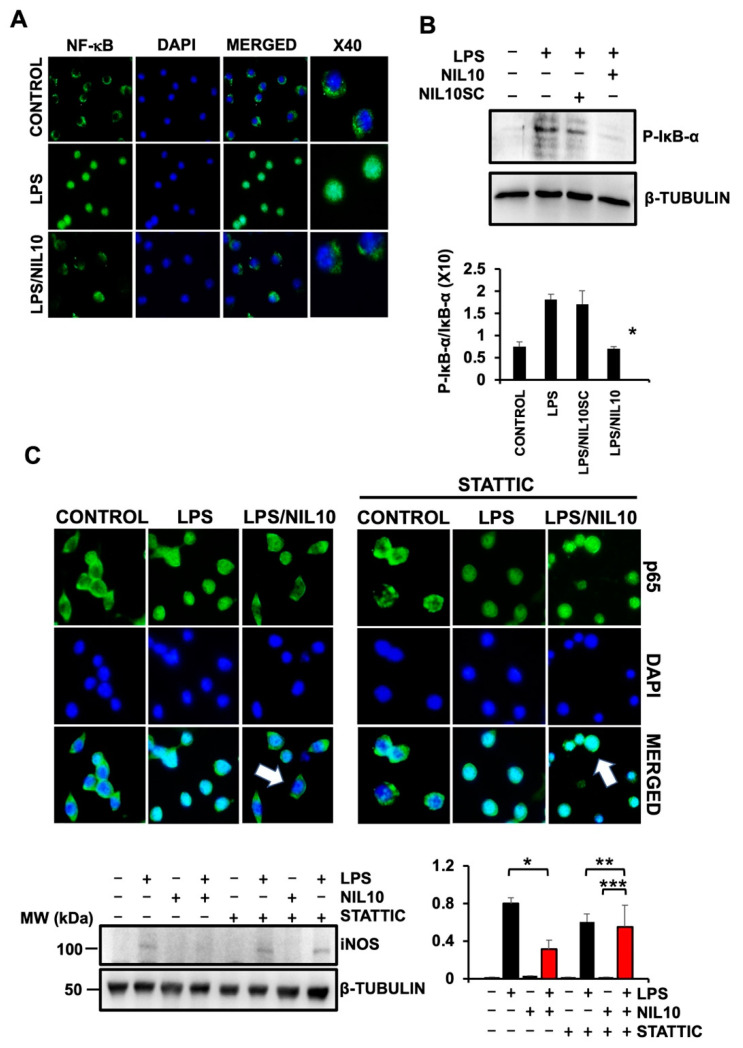
NIL10 prevents nuclear translocation of NF-ĸB through STAT3 activation in RAW 264.7 macrophages. (**A**). Confocal microscopy detection of NF-ĸB (p65) in RAW 264.7 cells stimulated with 500 µM LPS or in combination with NIL10. Nuclei were stained with DAPI. (**B**). Immunoblot detection of P- IĸB-α in RAW 264.7 cells treated with NIL10 or NIL10SC. (*n* = 3, results expressed as Mean ± SD. * *p* < 0.05 LPS/NIL10 vs. LPS/NIL10SC). (**C**). Confocal microscopy detection as in A, in which STATTIC, a pharmacological inhibitor of STAT3 was incubated. Arrows point to nuclear or cytoplasmic localization of p65. Lower panels: immunoblot detection of iNOS in cells as in C. (*n* = 3, results expressed as Mean ± SD. * *p* < 0.03 LPS vs. LPS/NIL10 ** *p* < 0.05 LPS/STATTIC vs. LPS/NIL10SC/STATTIC. *** *p* < 0.001 NIL10/STATIC vs. LPS/NIL10/STATIC).

The anti-inflammatory effect of NIL10 was further investigated in RAW 264.7 macrophages stimulated with 500 µM LPS. Nuclear translocation of pro-inflammatory NF-ĸB transcription factor was prevented by NIL10 (Figure 9A), at least by suppressing phosphorylation of the NF-κB cytoplasmic sequestering IĸB-α (Figure 9B), required for IĸB-α proteasome-mediated proteolytic degradation.

To further validate the anti-inflammatory effect of NIL10, we stimulated NF-ĸB nuclear translocation with 500 µM LPS in RAW 264.7 cells co-incubated with 5 µM of STAT3-specific pharmacological inhibitor STATTIC. This prevented the NIL10-induced cytoplasmic localization of NF-ĸB in LPS-treated cells, thus indicating that NIL10 inhibits NF-ĸB nuclear translocation at least, by promoting STAT3 activation (Figure 9C). The expression of the readout inducible nitric oxide synthase (iNOS) by pro-inflammatory signals was also assayed. As shown, iNOS was expressed by stimulating RAW 264.7 macrophages with 500 µM LPS and significantly inhibited by co-incubation with NIL10. The effect was reversed in the presence of STATTIC (Figure 9 lower panels). Taken together, NIL10 may act as an anti-inflammatory effector through the IL-10/STAT3 signaling pathway in myocardial ischemia/reperfusion.

## 4. Discussion

In the current work, we showed that NIL10, a novel anti-inflammatory nanoparticle, induces cardiac protection after acute myocardial infarction following cardiac ischemia/reperfusion. Administration of NIL10 24 h after IR attenuated interstitial fibrosis and improved LVEF in mice and pigs by days 3 and 7 after IR. The same pattern applied to IL-10 knockout mice, while in IL-10-receptor-deficient mice, NIL10 did not have an effect on cardiac recovery. As shown by confocal microscopy, NIL10 binds to IL-10R, inducing accumulation of M2-like macrophage populations after 3 days of IR, in which anti-inflammatory cytokines IL-4, IL-7, IL-10, IL-13, IL-16, and IL-27 were increased, at least through STAT-3-dependent inhibition of NF-κB nuclear translocation. 

IL-10 is an anti-inflammatory cytokine that leads to cardiovascular protection in human atherosclerosis [31], acute coronary syndrome [32], unstable angina [33], and heart failure [34]. IL-10 plays an important role in the final outcome of myocardial infarction. A positive correlation between serum IL-10 levels with LVEF in patients after myocardial infarction suggests the implication of IL-10 in predicting acute and chronic heart failure [35]. This is shown in patients with metabolic syndrome, in which higher IL-10 serum levels are associated with a lower incidence of severe coronary artery disease [32,35]. Here, we provide a novel compound based on the anti-inflammatory properties of IL-10, in which, both in mice and pigs, it has been shown to prevent cardiac necrosis after myocardial ischemia/reperfusion.

The described benefits of using small molecules, including oligopeptides, include acting as an agonist of several proteins. We previously described the cardioprotective effect of intravenous administration of NAP9. The nanoparticles carry a small peptide, which specifically binds to EMMPRIN, a protein involved in the extracellular matrix degradation during myocardial infarction in mice and pigs subjected to myocardial IR [36]. Others have found that the synthetic IL-10 analogue peptide, IT9302, can interact with IL-10 receptor and mimic its anti-inflammatory effects both in vitro in human melanoma cells [37] (in monocyte differentiation to TGF-β dendritic cells [26]) and in rabbits subjected to acute pancreatitis [38] and acute lung injury [39]. However, the potential benefits of IT9302 in other inflammatory conditions are poorly understood.

The use of several types of peptides in rodent models of myocardial infarction has been widely described. Examples include the use of Tat-DAXXp, a fusion peptide with Tat-cell-penetrating peptide and the death-associated protein peptide [40], a synthetic apolipoprotein A-I mimetic peptide 4F [41], the E-domain region of the human MGF protein [42], or UM206, a peptide derived from regions of high homology between Wnt proteins [43]. Each example is a promising strategy for preventing progression of disease in rodents, although no studies have yet been performed in large animal models prior to validating the strategy in patients suffering a myocardial infarction. In the current work, we provided the efficacy of using a new IT9302 conjugated nanoparticle, both in mice and in pigs, with similar results.

Currently, the efficacy of specific treatments after myocardial infarction is limited for reasons that include lack of specific biodistribution, reduced half-life, or even toxicity issues. Given their small size, instead of using antibodies, many therapeutic strategies have used nanoparticles conjugated with the therapeutic principle, enabling them to freely travel throughout the vasculature and reach the target faster. They are also biocompatible, with very little chance of rejection [44]. NIL10 is coated with PEGs (see methods for details), conferring a feature that implies a significant drug delivery (peptide IT9302 binding to IL10 receptor) to target cells and tissues with respect to empty peptide administration and decrease immunogenicity. Most importantly, PEG-coated NIL10 shields the surface from aggregation, phagocytosis, and degradation, thus prolonging systemic circulation time with respect to other molecules, including chemicals, peptides, or antibodies [45]. In addition, Z-potential and size ensured nanoparticle stability and tissular accessibility after IV administration. 

The use of nanoparticles is preferred over antibodies for the treatment of inflammatory diseases, in which eliciting immunostimulation is undesirable and should be minimized [46]. NIL10 activates the IL10 downstream signaling pathway by interacting with the IL10 receptor in vivo, while the use of anit-IL10 antibodies has the opposite effect, as shown when promoting collateral growth in mice subjected to hind limb ischemia. In this case, administration of recombinant IL10 induced reperfusion recovery, while incubation with anti-IL10 prevented the effect [20]. The use of anti-IL10 antibodies was also assayed in other inflammatory diseases, including psoriasis lesions; anti-IL10 antibody treatment upregulates IL-17A, which plays a central role in psoriasis pathogenesis [47]. Furthermore, nanoparticles are extremely versatile, as additional components can be incorporated into their composition to enable the acquisition of new functionalities. Such is the case of NAP9 [27,36] and NIL10—both are nanoparticles based on lipid micelles and incorporate a fluorochrome in their composition to gain visualization by fluorescence, conferring specificity.

Theranostics is defined as the combination of diagnostic and therapeutic capability on a single agent and, depending on their composition, nanoparticles are unique compounds suitable to be used as theranostic agents. Chemicals, antibodies, and peptides have been used to induce and/or inhibit several signaling pathways associated with the onset and progression of disease. However, very few compounds, including specific engineered nanoparticles, may have the ability to have a theranostic effect. As mentioned above, we engineered gadolinium containing NAP9 nanoparticles, revealing a theranostic effect against cardiac ischemia/reperfusion in mice and in pigs. NAP9 nanoparticles incorporate a small peptide, AP9, which specifically binds to EMMPRIN, an inflammatory molecule that induces MMP9 expression and activity in response to cardiac ischemia. NAP9 significantly improved cardiac function after IR and, thanks to the presence of gadolinium, NAP9 was visualized by MRI, correlating the extension of cardiac necrosis and healing overtime [27,36].

IL-10 triggers downstream signaling through binding to IL-10 receptors, which phosphorylate JAK1 and further activate the intracellular domain of IL-10R-a, required for the recruitment and phosphorylation of STAT3, which, at the end, regulates gene expression of anti-inflammatory genes [19]. Interestingly, others found that the IL-10 functional domain homolog IT9302 induced monocyte differentiation to TGF-β tolerogenic dendritic cells by an independent JNK/STAT3 signaling pathway [37]. However, our results point to STAT3 activation as a mechanism induced by NIL10 in cardiac protection against myocardial infarction. This apparent contradiction may lie in the mechanism of IT9302 administration, which, in the case of NIL10, specifically binds to IL-10 receptor, as shown by confocal microscopy when compared to its negative control, NIL10SC (Figure 1). Indeed, in IL-10 receptor null mice subjected to myocardial IR, administration of NIL10 had no effect, while in IL10 knockout animals, NIL10, but not NIL10SC, recovered cardiac protection, as detected by an improvement of LVEF in these mice (Figure 3).

The anti-inflammatory effect of IL-10 is in addition driven by STAT3-mediated induction of several mediators, including certain transcription factors, such as ETV3 and the Strawberry notch homologue-2, which inhibit NF-ĸB-mediated transcription in LPS-stimulated macrophages [30]. Here, we also found that NIL10 induced STAT3-NF-κB inhibition in LPS-stimulated RAW 264.7 macrophages, as confirmed by the reversion of NIL10-mediated inhibition of iNOS expression in LPS-stimulated cells by pharmacological inhibition of STAT-3 in this context.

## 5. Conclusions

In conclusion, we provided a feasible delivery of a novel compound to target the inflammatory response after acute myocardial infarction and reperfusion, representing a reliable strategy to preserve heart contractility, as a mechanism to prevent the progression of myocardial necrosis. In addition to NIL10, our approach supports the use of additional peptides against specific targets susceptible to inhibition in the acute phase of heart failure postinfarction. Further studies to test the long-term effects of NIL10 administration in pigs will be a key step to initiating validation in the clinical setting.

## Data Availability

Not applicable.

## Supplementary Materials

The following supporting information can be downloaded at: https://www.mdpi.com/article/10.3390/pharmaceutics14102044/s1, Figure S1: Dose-effect of NIL10 on animal survival and organ biodistribution; Figure S2: Kaplan Meier curve; Figure S3: Cytokine array used in the study; Figure S4: Gating strategy used to identify macrophage-cell subsets in the healthy and infarcted mice heart. A sequential gating strategy was first used to identify the M2 population expressing specific macrophage marker CD68, followed by the identification of the population with overlapping expression patterns (CD68/CD206).

## References

[1] Virani S.S., Alonso A., Benjamin E.J., Bittencourt M.S., Callaway C.W., Carson A.P., Chamberlain A.M., Chang A.R., Cheng S., Delling F.N. (2020). Heart disease and stroke statistics—2020 update: A report from the American Heart Association. Circulation.

[2] Velagaleti R.S., Pencina M.J., Murabito J.M., Wang T.J., Parikh N.I., D’Agostino R.B., Levy D., Kannel W.B., Vasan R.S. (2008). Long-term trends in the incidence of heart failure after myocardial infarction. Circulation.

[3] Chen J., Hsieh A.F.C., Dharmarajan K., Masoudi F.A., Krumholz H.M. (2013). National trends in heart failure hospitalization after acute myocardial infarction for medicare beneficiaries 1998–2010. Circulation.

[4] Ezekowitz J.A., Kaul P., Bakal J.A., Armstrong P.W., Welsh R.C., McAlister F.A. (2009). Declining In-Hospital Mortality and Increasing Heart Failure Incidence in Elderly Patients with First Myocardial Infarction. J. Am. Coll. Cardiol..

[5] Prabhu S.D., Frangogiannis N.G. (2016). The biological basis for cardiac repair after myocardial infarction. Circ. Res..

[6] Frangogiannis N. (2006). Targeting the Inflammatory Response in Healing Myocardial Infarcts. Curr. Med. Chem..

[7] Westman P.C., Lipinski M.J., Luger D., Waksman R., Bonow R.O., Wu E., Epstein S.E. (2016). Inflammation as a Driver of Adverse Left Ventricular Remodeling after Acute Myocardial Infarction. J. Am. Coll. Cardiol..

[8] Peet C., Ivetic A., Bromage D.I., Shah A.M. (2020). Cardiac monocytes and macrophages after myocardial infarction. Cardiovasc. Res..

[9] Ramirez-Carracedo R., Tesoro L., Hernandez I., Diez-Mata J., Piñeiro D., Hernandez-Jimenez M., Zamorano J.L., Zaragoza C. (2020). TargetingTLR4 with aptoll improves heart function in response to coronary ischemia reperfusion in pigs undergoing acute myocardial infarction. Biomolecules.

[10] Fujiu K., Wang J., Nagai R. (2014). Cardioprotective function of cardiac macrophages. Cardiovasc. Res..

[11] Martinez F.O., Gordon S. (2014). The M1 and M2 paradigm of macrophage activation: Time for reassessment. F1000Prime Rep..

[12] Sica A., Mantovani A. (2012). Macrophage plasticity and polarization: In vivo veritas. J. Clin. Investig..

[13] Murray P.J., Allen J.E., Biswas S.K., Fisher E.A., Gilroy D.W., Goerdt S., Gordon S., Hamilton J.A., Ivashkiv L.B., Lawrence T. (2014). Macrophage Activation and Polarization: Nomenclature and Experimental Guidelines. Immunity.

[14] Josephson K., Logsdon N.J., Walter M.R. (2001). Crystal structure of the IL-10/IL-10R1 complex reveals a shared receptor binding site. Immunity.

[15] Tedgui A., Mallat Z. (2001). Anti-inflammatory mechanisms in the vascular wall. Circ. Res..

[16] Van Der Meeren A., Squiban C., Gourmelon P., Lafont H., Gaugler M.H. (1999). Differential regulation by IL-4 and IL-10 of radiation-induced IL-6 and IL-8 production and ICAM-1 expression by human endothelial cells. Cytokine.

[17] Selzman C.H., McIntyre R.C., Shames B.D., Whitehill T.A., Banerjee A., Harken A.H. (1998). Interleukin-10 inhibits human vascular smooth muscle proliferation. J. Mol. Cell. Cardiol..

[18] Weber-Nordt R.M., Riley J.K., Greenlund A.C., Moore K.W., Darnell J.E., Schreiber R.D. (1996). Stat3 recruitment by two distinct ligand-induced, tyrosine- phosphorylated docking sites in the interleukin-10 receptor intracellular domain. J. Biol. Chem..

[19] Riley J.K., Takeda K., Akira S., Schreiber R.D. (1999). Interleukin-10 Receptor Signaling through the JAK-STAT Pathway. J. Biol. Chem..

[20] Götze A.M., Schubert C., Jung G., Dörr O., Liebetrau C., Hamm C.W., Schmitz-Rixen T., Troidl C., Troidl K. (2020). IL10 Alters Peri-Collateral Macrophage Polarization and Hind-Limb Reperfusion in Mice after Femoral Artery Ligation. Int. J. Mol. Sci..

[21] Zaringhalam J., Akhtari Z., Eidi A., Ruhani A.H., Tekieh E. (2014). Relationship between serum IL10 level and p38MAPK enzyme activity on behavioral and cellular aspects of variation of hyperalgesia during different stages of arthritis in rats. Inflammopharmacology.

[22] Qian Q., Wu C., Chen J., Wang W. (2020). Relationship between IL10 and PD-L1 in Liver Hepatocellular Carcinoma Tissue and Cell Lines. BioMed Res. Int..

[23] Hsu T.I., Wang Y.C., Hung C.Y., Yu C.H., Su W.C., Chang W.C., Hung J.-J. (2016). Positive feedback regulation between IL10 and EGFR promotes lung cancer formation. Oncotarget.

[24] McKelvey R., Berta T., Old E., Ji R.R., Fitzgerald M. (2015). Neuropathic pain is constitutively suppressed in early life by anti-inflammatory neuroimmune regulation. J. Neurosci..

[25] López M.N., Pesce B., Kurte M., Pérez C., Segal G., Roa J., Aguillón J.C., Mendoza-Naranjo A., Gesser B., Larsen C. (2011). A synthetic peptide homologous to IL-10 functional domain induces monocyte differentiation to TGF-β+ tolerogenic dendritic cells. Immunobiology.

[26] Yurchenko V., Pushkarsky T., Li J.H., Dai W.W., Sherry B., Bukrinsky M. (2005). Regulation of CD147 cell surface expression: Involvement of the proline residue in the CD147 transmembrane domain. J. Biol. Chem..

[27] Ramirez-Carracedo R., Sanmartin M., Ten A., Hernandez I., Tesoro L., Diez-Mata J., Botana L., Ovejero-Paredes K., Filice M., Alberich-Bayarri A. (2022). Theranostic Contribution of Extracellular Matrix Metalloprotease Inducer-Paramagnetic Nanoparticles Against Acute Myocardial Infarction in a Pig Model of Coronary Ischemia-Reperfusion. Circ. Cardiovasc. Imaging.

[28] Babicki S., Arndt D., Marcu A., Liang Y., Grant J.R., Maciejewski A., Wishart D.S. (2016). Heatmapper: Web-enabled heat mapping for all. Nucleic Acids Res..

[29] Shimamoto A., Chong A.J., Yada M., Shomura S., Takayama H., Fleisig A.J., Agnew M.L., Hampton C.R., Rothnie C.L., Spring D.J. (2006). Inhibition of Toll-like receptor 4 with eritoran attenuates myocardial ischemia-reperfusion injury. Circulation.

[30] Saraiva M., Vieira P., O’Garra A. (2020). Biology and therapeutic potential of interleukin-10. J. Exp. Med..

[31] Ambrosius W., Kazmierski R., Michalak S., Kozubski W. (2006). Anti-inflammatory cytokines in subclinical carotid atherosclerosis. Neurology.

[32] Heeschen C., Dimmeler S., Hamm C.W., Fichtlscherer S., Boersma E., Simoons M.L., Zeiher A.M. (2003). CAPTURE Study Investigators. Serum level of the antiinflammatory cytokine interleukin-10 is an important prognostic determinant in patients with acute coronary syndromes. Circulation.

[33] Smith D.A., Irving S.D., Sheldon J., Cole D., Kaski J.C. (2001). Serum levels of the antiinflammatory cytokine interleukin-10 are decreased in patients with unstable angina. Circulation.

[34] Miettinen K.H., Lassus J., Harjola V.P., Siirilä-Waris K., Melin J., Punnonen K.R., Nieminen M.S., Laakso M., Peuhkurinen K.J. (2008). Prognostic role of pro- and anti-inflammatory cytokines and their polymorphisms in acute decompensated heart failure. Eur. J. Heart Fail..

[35] Lakhani H.V., Khanal T., Gabi A., Yousef G., Alam M.B., Sharma D., Aljoudi H., Puri N., Thompson E., Shapiro J.I. (2018). Developing a panel of biomarkers and miRNA in patients with myocardial infarction for early intervention strategies of heart failure in West Virginian population. PLoS ONE.

[36] Cuadrado I., Piedras M.J., Herruzo I., Turpin Mdel C., Castejón B., Reventun P., Martin A., Saura M., Zamorano J.L., Zaragoza C. (2016). EMMPRIN-Targeted magnetic nanoparticles for in vivo visualization and regression of acute myocardial infarction. Theranostics.

[37] Kurte M., López M., Aguirre A., Escobar A., Aguillón J.C., Charo J., Larsen C.G., Kiessling R., Salazar-Onfray F. (2004). A synthetic peptide homologous to functional domain of human IL-10 down-regulates expression of MHC class I and Transporter associated with Antigen Processing 1/2 in human melanoma cells. J. Immunol..

[38] Osman M.O., Gesser B., Mortensen J.T., Matsushima K., Jensen S.L., Larsen C.G. (2002). Profiles of pro-inflammatory cytokines in the serum of rabbits after experimentally induced acute pancreatitis. Cytokine.

[39] Osman M.O., Jacobsen N.O., Kristensen J.U., Deleuran B., Gesser B., Larsen C.G., Jensen S.L. (1998). IT 9302, a synthetic interleukin-10 agonist, diminishes acute lung injury in rabbits with acute necrotizing pancreatitis. Surgery.

[40] Boisguérin P., Covinhes A., Gallot L., Barrère C., Vincent A., Busson M., Piot C., Nargeot J., Lebleu B., Barrère-Lemaire S. (2020). A novel therapeutic peptide targeting myocardial reperfusion injury. Cardiovasc. Res..

[41] Moreira R.S., Irigoyen M.C., Capcha J.M.C., Sanches T.R., Gutierrez P.S., Garnica M.R., Noronha I.d., Andrade L. (2020). Synthetic apolipoprotein A-I mimetic peptide 4F protects hearts and kidneys after myocardial infarction. Am. J. Physiol. Regul. Integr. Comp. Physiol..

[42] Peña J.R., Pinney J.R., Ayala P., Desai T.A., Goldspink P.H. (2015). Localized delivery of mechano-growth factor E-domain peptide via polymeric microstructures improves cardiac function following myocardial infarction. Biomaterials.

[43] Laeremans H., Hackeng T.M., van Zandvoort M.A., Thijssen V.L., Janssen B.J., Ottenheijm H.C.J., Smits J.F.M., Blankesteijn W.M. (2011). Blocking of frizzled signaling with a homologous peptide fragment of wnt3a/wnt5a reduces infarct expansion and prevents the development of heart failure after myocardial infarction. Circulation.

[44] MacRitchie N., Di Francesco V., Ferreira M.F.M.M., Guzik T.J., Decuzzi P., Maffia P. (2021). Nanoparticle theranostics in cardiovascular inflammation. Semin. Immunol..

[45] Suka J.S., Xua O., Kima N., Hanesa J., Ensigna L.M. (2016). PEGylation as a strategy for improving nanoparticle-based drug and gene delivery. Adv. Drug. Deliv. Rev..

[46] Zolnik B.S., González-Fernández A., Sadrieh N., Dobrovolskaia M.A. (2010). Nanoparticles and the Immune System. Endocrinology.

[47] Xu X., Prens E., Florencia E., Leenen P., Boon L., Asmawidjaja P., Mus A.M., Lubberts E. (2021). Interleukin-17A Drives IL-19 and IL-24 Expression in Skin Stromal Cells Regulating Keratinocyte Proliferation. Front. Immunol..

