# Supplementary material for: NIL10: A New IL10-Receptor Binding Nanoparticle That Induces Cardiac Protection in Mice and Pigs Subjected to Acute Myocardial Infarction through STAT3/NF-κB Activation
Source: Pharmaceutics. 2022 Sep 26;14(10):2044. doi: 10.3390/pharmaceutics14102044 (PMC9608724; doi:10.3390/pharmaceutics14102044)
Supplement: Supplementary file 1 [file pharmaceutics-14-02044-s001.zip › pharmaceutics-1825235-supplementary.pdf]

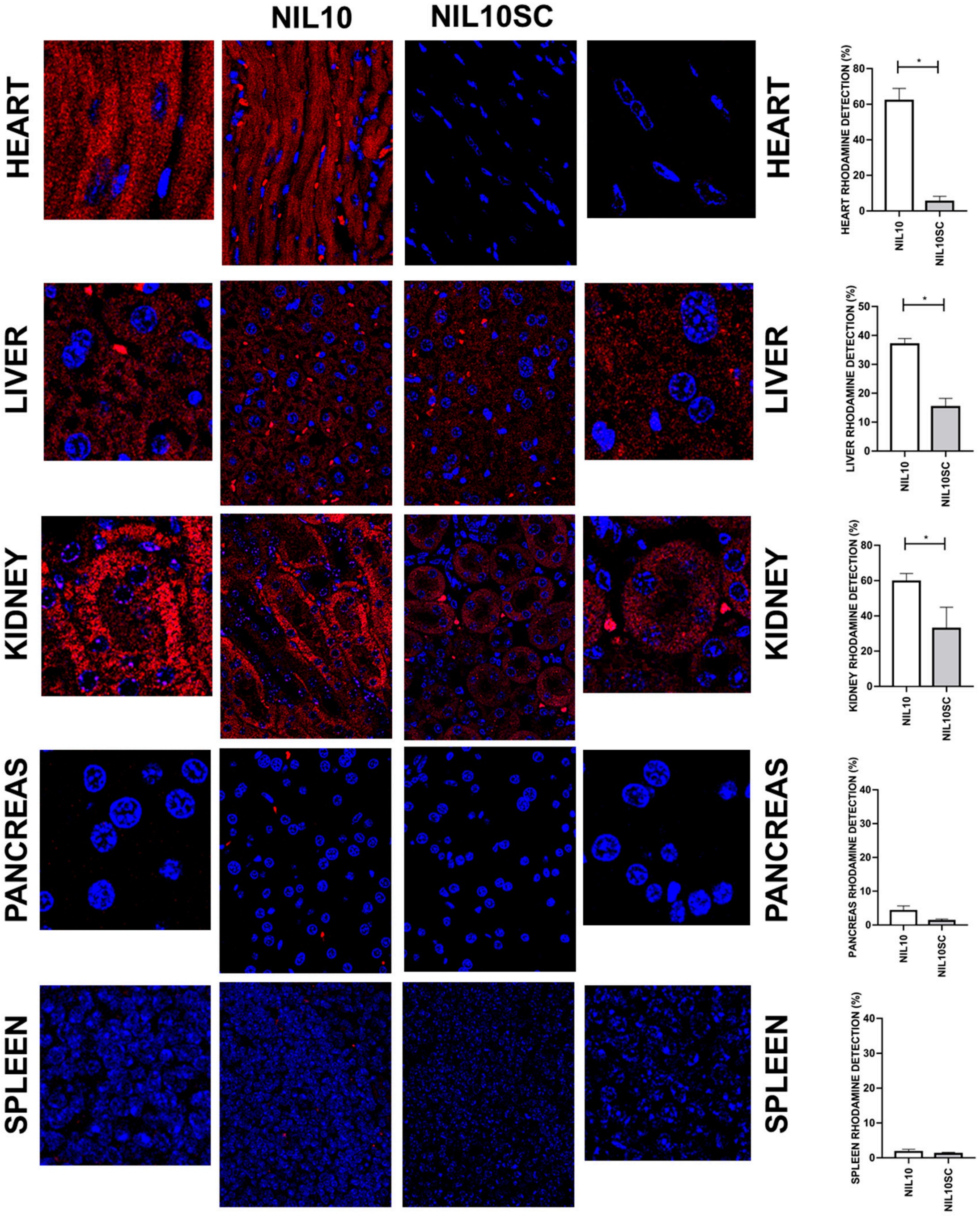

Supplementary Figure S1. Biodistribution of NIL10 and NIL10SC. Confocal microscopy detection of 1 mg/kg NIL10 and NIL10SC in the heart, liver, kidney pancreas, and spleen of healthy animals by day 7 after IV injection (N=10 mice/group).

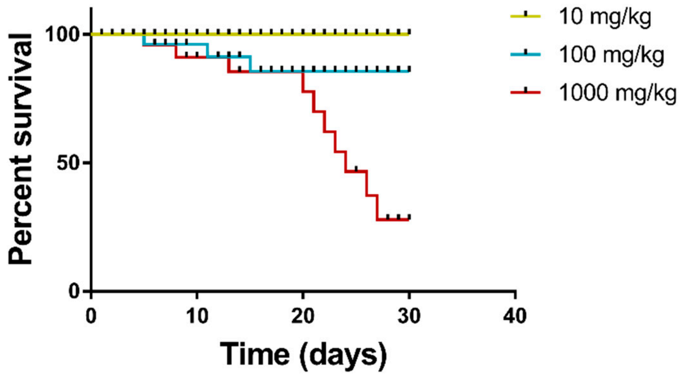

Supplementary Figure S2. Kaplan Meier curve showing the percentage of survival of healthy mice injected with the doses indicated of NIL10 (N=5mice/condition).

Inflammatory	Anti-inflammatory	Chemokines
C5a	IL1-RA	CXCL1
G-CSF	IL-4	CXCL2
GM-CSF	IL-5	CXCL3
CCL1	IL-7	CXCL9
CCL11	IL-10	CXCL10
sICAM-1	IL-11	CXCL11
IFN-gamma	IL-13	CXCL12
IL1-alpha	IL-16	CCL2
IL1-beta	IL27	CCL3
IL-2		CCL4
IL-3		CCL5 (RANTES)
IL-6		CCL12
IL12-P70		CCL17
IL-17		
IL-23		
TNF-ALPHA		
TREM-1		
MCSF		

Suppelemtary Figure S3. Cytokine array used in the study.

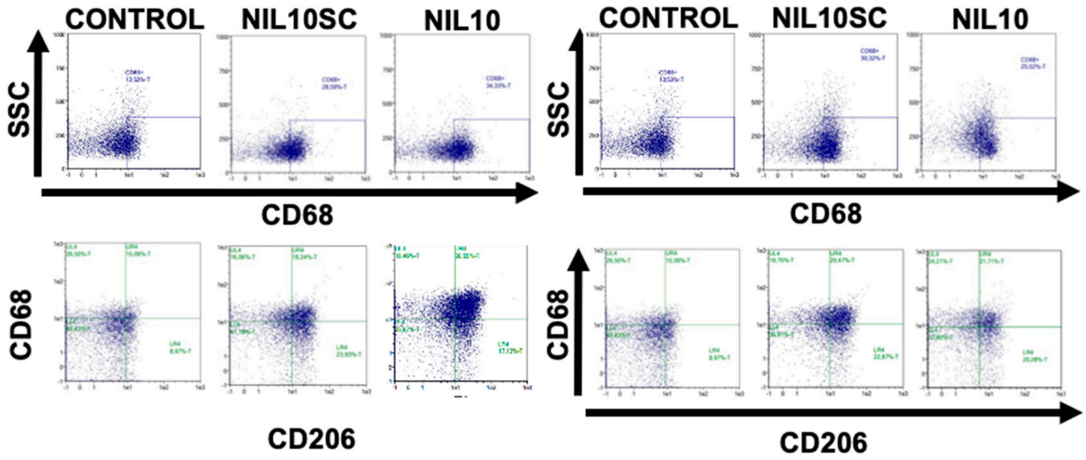

Supplementary Figure S4. Gating strategy used to identify macrophage-cell subsets in the healthy and infarcted mice heart. A sequential gatting strategy was first used to identify M2 population expressing specific macrophage marker CD68, followed by the identification of the population with overlapping expression patterns (CD68/CD206).
